# The glucocorticoid mometasone furoate is a novel FXR ligand that decreases inflammatory but not metabolic gene expression

**DOI:** 10.1038/srep14086

**Published:** 2015-09-15

**Authors:** Ingrid T. G. W. Bijsmans, Chiara Guercini, José M. Ramos Pittol, Wienand Omta, Alexandra Milona, Daphne Lelieveld, David A. Egan, Roberto Pellicciari, Antimo Gioiello, Saskia W. C. van Mil

**Affiliations:** 1 Center for Molecular Medicine, UMC Utrecht, Utrecht, the Netherlands; 2 TES Pharma, Loc. Taverne, Corciano (Perugia), Italy; 3 Cell Screening Core, Department of Cell Biology, UMC Utrecht, Utrecht, the Netherlands; 4 Department of Pharmaceutical Sciences, University of Perugia, Perugia, Italy

## Abstract

The Farnesoid X receptor (FXR) regulates bile salt, glucose and cholesterol homeostasis by binding to DNA response elements, thereby activating gene expression (direct transactivation). FXR also inhibits the immune response via tethering to NF-κB (tethering transrepression). FXR activation therefore has therapeutic potential for liver and intestinal inflammatory diseases. We aim to identify and develop gene-selective FXR modulators, which repress inflammation, but do not interfere with its metabolic capacity. In a high-throughput reporter-based screen, mometasone furoate (MF) was identified as a compound that reduced NF-κB reporter activity in an FXR-dependent manner. MF reduced mRNA expression of pro-inflammatory cytokines, and induction of direct FXR target genes in HepG2-GFP-FXR cells and intestinal organoids was minor. Computational studies disclosed three putative binding modes of the compound within the ligand binding domain of the receptor. Interestingly, mutation of W469A residue within the FXR ligand binding domain abrogated the decrease in NF-κB activity. Finally, we show that MF-bound FXR inhibits NF-κB subunit p65 recruitment to the DNA of pro-inflammatory genes *CXCL2* and *IL8*. Although MF is not suitable as selective anti-inflammatory FXR ligand due to nanomolar affinity for the glucocorticoid receptor, we show that separation between metabolic and anti-inflammatory functions of FXR can be achieved.

Nuclear receptors (NRs) are ligand-activated transcription factors regulating a large variety of target genes^1^. A variety of molecular mechanisms by which NRs regulate transcription have been identified (reviewed elsewhere, see^2^,^3^). Classically, NRs directly bind to consensus response elements in target genes, thereby either activating or repressing transcription. In addition, NRs function independently of DNA binding, by tethering to other transcription factors (e.g. NF-κB, AP-1, or other NRs^2^). Since NR activity is regulated by specific ligands which can easily pass cell membranes, NRs are ideal drug targets. However, serious side-effects of the current NR drugs limit their utility due to activation of all transcriptional NR actions^4^. Therefore, many studies are undertaken to develop selective NR ligands^5^.

Farnesoid X receptor (FXR, also known as NR1H4) is a nuclear receptor activated by endogenous bile acids (BAs). Upon activation, FXR heterodimerizes with retinoid X receptor (RXR, also known as NR2B1) and binds FXR responsive elements in the promoters of target genes, leading to dissociation of the co-repressor complex, recruitment of co-activators, and transcription initiation. In this manner, FXR regulates bile salt concentrations in liver and intestinal cells by regulating bile salt transport systems, bile salt metabolism, and *de novo* synthesis (via *SHP/FGF19* upregulation) from cholesterol in the liver^6^. FXR also regulates glucose and fat homeostasis via direct binding to target genes^7^,^8^. We and others have shown that FXR also functions in a DNA-independent manner, by binding to NF-κB, thereby inhibiting NF-κB activity. This results in decreased pro-inflammatory cytokine expression in both liver and intestine^9^,^10^,^11^. FXR activation by a full agonist, obeticholic acid (OCA, 6-ECDCA), strongly improved clinical symptoms and histology of dextran sodium sulphate (DSS)- and trinitrobenzene sulphonic acid (TNBS)-induced colitis in wild type (WT), but not in *FXR*−/− mice. In addition, intestinal epithelial permeability was decreased and pro-inflammatory cytokine mRNA expression was inhibited upon FXR activation^9^. This provides a clear rationale for further exploration of the use of FXR agonists as novel therapeutics for chronic inflammation of liver and intestine. In this context, the development of gene-selective FXR modulators, referred to as SBARMs (selective bile acid receptor modulators), is particularly sought in view of their ability to modulate specific genes without affecting others, thus limiting potential side-effects of full FXR agonism upon chronic treatment. We therefore aim to develop selective anti-inflammatory FXR agonists, able to selectively interfere with the two molecular mechanisms by which FXR regulates metabolism and inflammation.

The previously reported high-throughput screening methods to identify FXR agonists are not suitable to detect anti-inflammatory FXR ligands. Co-factor recruitment assays, or comparable assays monitor the recruitment of a co-activator peptide upon ligand binding^12^,^13^, but this is not anticipated to happen when FXR tethers to NF-κB to inhibit its activity. It is expected that FXR recruits a co-repressor complex in this situation, as has been shown recently for FXR^14^ and for other nuclear receptors^15^. For that reason, we developed an automated high-throughput luciferase reporter assay to screen chemical libraries to identify compounds that decrease NF-κB activity in an FXR-dependent manner. Mometasone furoate (MF) was identified to regulate NF-κB activity, but not metabolic target genes, in the presence, but not in the absence of FXR, suggesting that separation of FXR anti-inflammatory actions from its metabolic actions is achievable by selective agonists.

## Results

### Luciferase reporter screen identifies five compounds decreasing TNFα-induced NF-κB activity

**Figure 1:**
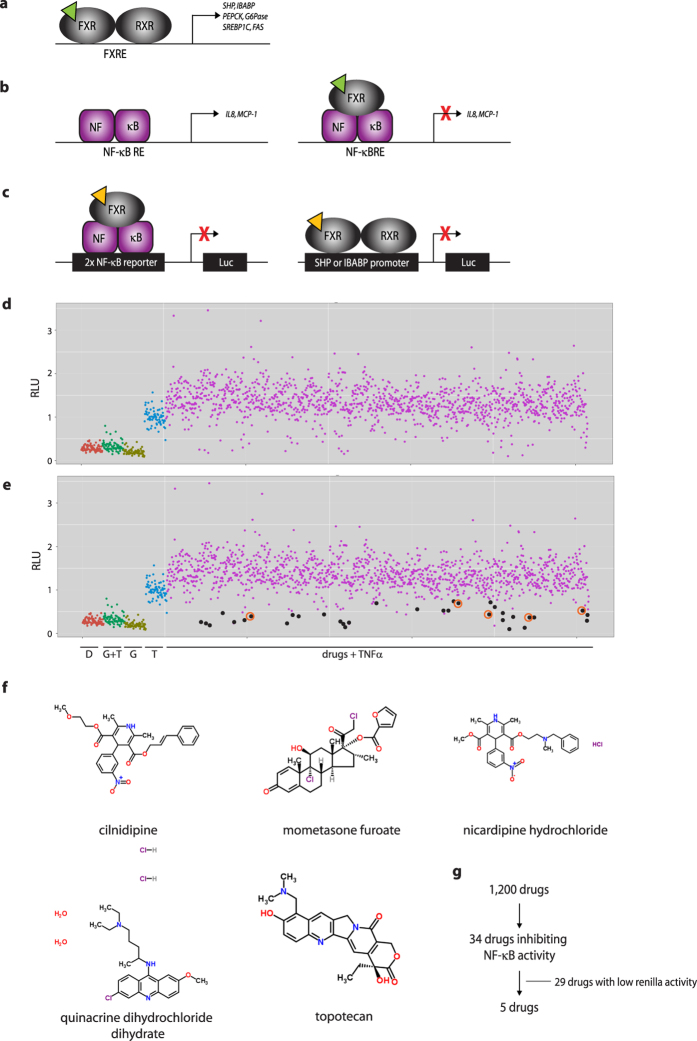
Luciferase reporter assay identifies 5 compounds decreasing TNFα-induced NF-κB transcriptional activity. Schematic representation of the molecular FXR actions in regulation of bile salt, glucose and fat metabolism via direct DNA binding (**A**), and ameliorating inflammation via tethering transrepression of NF-κB (**B**). Ligand (green triangle) activated FXR binds to FXR responsive elements (FXREs), thereby activating target genes involved in bile salt homeostasis (*SHP*, *IBABP*), glucose (*PEPCK*, *G6Pase*), and fat metabolism (*SREBP1C*, *FAS*). Binding of NF-κB to its responsive element (NF-κB RE) results in expression of pro-inflammatory cytokines, such as *IL8* and *MCP-1*. FXR binding to NF-κB inhibits this activity, thereby decreasing pro-inflammatory cytokine expression. We have set up an automated high-throughput NF-κB luciferase reporter assay to test FXR-dependent reduction of NF-κB activity (**C**). We screened the Prestwick library containing 1,200 FDA approved drugs (yellow triangle) using this assay (left panel). Candidate drugs were subsequently tested for *IBABP* and *SHP* transactivation. Figures D-G depict hit selection. Figures **D and E** show the overall view of the screen. Indicated with black dots are the 34 drugs reducing TNFα-induced NF-κB transcriptional activity significantly (p < 0.05) (**E**). Low renilla values were considered to reflect poor transfection efficiency or cytotoxicity and were therefore eliminated, leaving 5 compounds significantly reducing NF-κB activity (indicated with black dots surrounded by orange circles. D: DMSO; G+T: GW4064 + TNFα; G: GW4064; T: TNFα (**E**). Figure **F** depicts the chemical structures of the five candidate compounds. Flowchart of hit selection is shown in (**G**).

In Fig. 1, we schematically depict the current knowledge on the mechanisms by which FXR regulates metabolism (Fig. 1A) and anti-inflammatory (Fig. 1B) effects. We hypothesize that ligands might be able to separate FXR metabolic from anti-inflammatory functions because of the different mechanisms of direct versus tethered DNA binding. In order to identify FXR-dependent anti-inflammatory ligands, we set up an automated high-throughput luciferase reporter assay to monitor NF-κB activity, and used it to screen the Prestwick Chemical Library®. Ideally, the ligands repress NF-κB activity (Fig. 1C, left panel), but do not induce SHP or IBABP transcriptional activity (Fig. 1C, right panel). HEK293T cells transfected with a NF-κB reporter construct in combination with FXR and RXR expression plasmids were incubated with TNFα to activate NF-κB. Of the 1,200 tested drugs (Fig. 1D; depicted in purple), 34 drugs inhibited TNFα-induced transcriptional activity of the NF-κB reporter (Fig. 1E, depicted in black). Drugs showing low renilla activity (suggesting compound cytotoxicity and/or low transfection efficiency) were excluded, yielding 4 candidate drugs significantly reducing NF-κB transcriptional activity (Fig. 1E, orange circles). Although nicardipine hydrochloride did not significantly reduce NF-κB activity in the primary screen, we analyzed this compound further since it is structurally related to cilnidipine (a statistical significant hit), and nicardipine hydrochloride was recently shown to function as an FXR agonist^16^. Chemical structures of the candidate compounds are depicted in Fig. 1F. Taken together, five compounds decreased TNFα-induced NF-κB activity (Fig. 1G).

### Mometasone furoate is an FXR modulator with predominant anti-inflammatory activity

**Figure 2:**
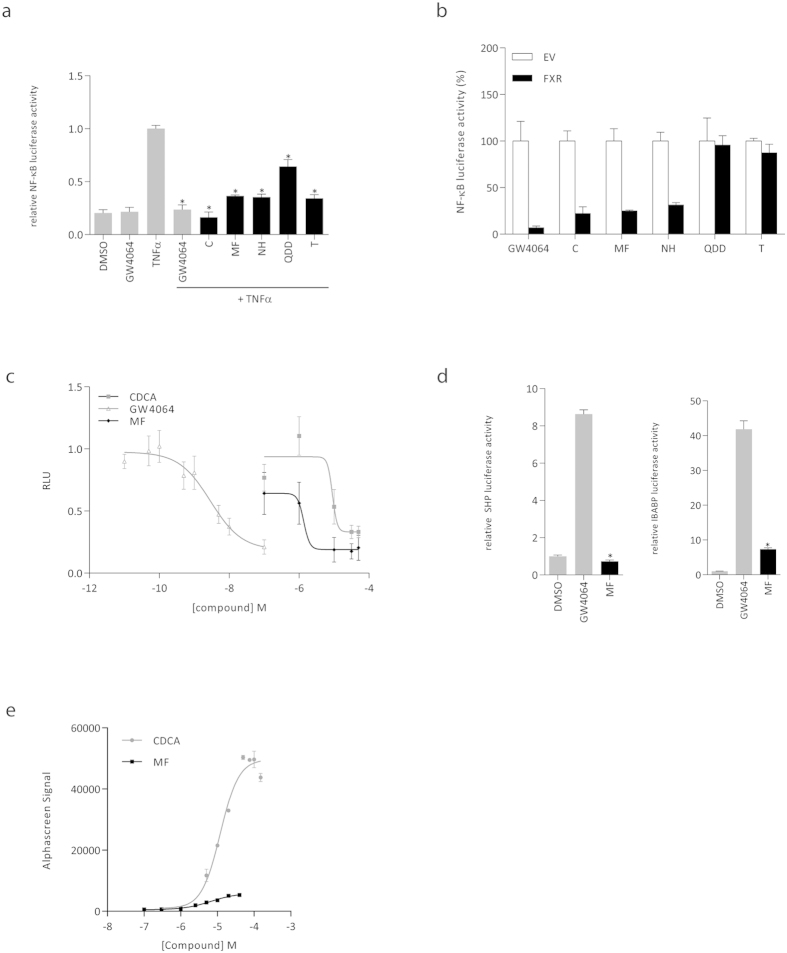
MF is an anti-inflammatory FXR modulator. (**A**) Validation of NF-κB luciferase reporter assay. HEK293T cells transfected with NF-κB reporter, expression plasmids for FXR and RXR, and pTK-renilla construct were treated with DMSO, GW4064 (1 μM), TNFα (5 ng/ml), GW4064 plus TNFα, or the indicated compounds (10 μM) in the presence of TNFα, for 24 hours. Cilnidipine (C), mometasone furoate (MF), nicardipine hydrochloride (NH), quinacrine dihydrochloride dehydrate (QDD), and topotecan (T) significantly reduced TNFα-induced NF-κB activity. The reporter assay was performed in quadruplicate in three independent experiments. Each bar represents mean ± SD of one representative experiment. *p < 0.001 as compared to TNFα treated cells. (**B**) FXR-dependent reduction of NF-κB transcriptional activity. The assay in (**A**) was repeated with empty vector (EV; white bars) and FXR overexpressing cells (black bars). Data are normalized to the EV activity for each compound. (**C**) Dose-response curve. MF treatment reduced TNFα-induced NF-κB activity in a dose-dependent manner, with an IC_50_ value of 1.4 μM. IC_50_ values of CDCA and GW4064 are 7.9 μM and 3.5 nM respectively. (**D**) Transactivation reporter assay SHP (left panel) and IBABP promoters (right panel). HEK293T cells transfected with SHP or IBABP promoter constructs, FXR and RXR, and renilla, were treated with DMSO, 1 μM GW4064, or 10 μM MF for 24 hours. Data presented show one representative experiment of 4 performed experiments. Each bar represents mean ± SD. *p < 0.001 compared to GW4064 treated cells. (**E**) FXR coactivator recruitment assay (AlphaScreen). Ligand binding domain of FXR (FXR-LBD) was incubated with increasing amounts of MF or CDCA to examine SRC-1 recruitment. Assay performed in triplicate. One representative experiment is shown.

In Fig. 2A we validated that the five compounds identified in our screen reduced NF-κB activity in a separate reporter assay. To investigate whether these 5 compounds repress NF-κB activity in an FXR-dependent manner, reporter assays were repeated comparing empty vector (pcDNA3.1) with FXR transfected cells. Three compounds, cilnidipine (C), mometasone furoate (MF), and nicardipine hydrochloride (NH) significantly decreased NF-κB activity in FXR transfected cells. Quinacrine dihydrochloride dihydrate (QDD) and topotecan (T) decreased NF-κB activity independent of FXR, possibly via binding to other nuclear receptors (Fig. 2B). C and NH were recently described to have transactivation activity^16^, and would thus also affect FXR metabolic function, therefore, upcoming experiments were performed for MF only. Figure 2C shows that NF-κB activity was reduced in a dose-dependent manner upon MF treatment. In conclusion, our screen revealed MF as a compound that has FXR-dependent anti-inflammatory properties.

Since we aim to develop selective anti-inflammatory FXR agonists, we next determined the capacity of MF to induce transcription of target genes via direct DNA binding to SHP and IBABP promoters. HEK293T cells were transfected with FXR, RXR, and either a SHP or IBABP promoter reporter construct. GW4064 induced a strong response for both promoters (9 and 42 fold respectively). MF showed no SHP, and a strongly reduced IBABP promoter activity compared to GW4064 (Fig. 2D). In addition, we characterized the binding potency of MF as ligand for FXR by performing an FXR-coactivator recruitment assay. In this assay, ligand binding induces the recruitment of the co-activator SRC-1 to the FXR ligand binding domain (LBD). MF appears a partial agonist with an EC_50_ of 10.9 ± 3.8 μM and efficacy of 12% compared to CDCA (Fig. 2E). In summary, GW4064 both activates transcription of SHP and IBABP promoters and inhibits transcription of the NF-κB promoter. In contrast, MF inhibits NF-κB activity comparable to GW4064 and CDCA, with low or absent activity on SHP and IBABP promoters.

### Mometasone furoate reduces endogenous pro-inflammatory gene expression in HepG2 cells and intestinal organoids in an FXR-dependent manner

**Figure 3:**
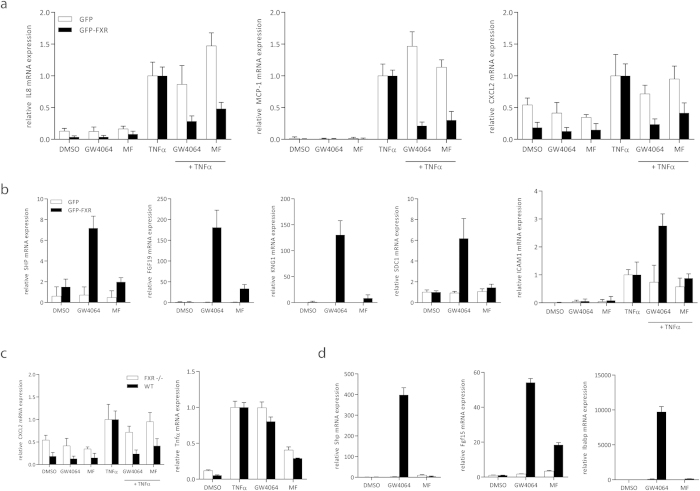
MF reduces pro-inflammatory gene expression in HepG2 cells and intestinal organoids. Endogenous FXR target gene expression in HepG2 cells stably overexpressing GFP (HepG2-GFP; white bars) or GFP-FXR (HepG2-GFP-FXR; black bars). (**A**) Cells were treated in triplicate with DMSO, 1 μM GW4064, 10 μM MF, 5 ng/ml TNFα, GW4064 plus TNFα, or TNFα plus MF for 24 hours. *IL8, MCP-1, and CXCL2* mRNA expression was analyzed by qRT-PCR in duplicate. (**B**) HepG2-GFP and HepG2-GFP-FXR cells were treated with DMSO, 1 μM GW4064 or 10 μM MF in triplicate for 24 hours. *SHP, FGF19, KNG1, SDC1 and ICAM1* mRNA expression was analyzed by qRT-PCR in duplicate. Each bar represents mean ± SD. (**C**,**D**) Small intestine derived organoids from 3WT and FXR−/− mice were treated with DMSO, 1 μM GW4064, 10 μM MF, 5 ng/ml TNFα, GW4064 plus TNFα, or TNFα plus MF for 24 hours. mRNA expression of each organoid line was analyzed by qRT-PCR in duplicate. Each bar represents mean ± SEM.

To extend the finding that MF reduced the NF-κB activity in an FXR dependent manner, we analyzed endogenous FXR and NF-κB target gene expression in HepG2 cells stably overexpressing GFP (HepG2-GFP) or GFP-FXR (HepG2-GFP-FXR). Cells were stimulated with TNFα to induce NF-κB activity. Indeed, NF-κB target genes *IL8, MCP-1,* and *CXCL2* increased upon TNFα stimulation. Co-stimulation with MF or GW4064 abolished this effect in HepG2-GFP-FXR cells but not in HepG2-GFP cells (Fig. 3A), indicating that FXR activation by GW4064 or MF blocks NF-κB activity. Direct FXR target genes *SHP, FGF19, KNG1, SDC1* and *ICAM1*^6^ mRNA expression was induced upon GW4064 treatment, however, only a minor increase was detected in HepG2-GFP-FXR cells treated with MF (Fig. 3B). To test whether MF also selectively affects pro-inflammatory gene expression in a model system closer to the *in vivo* situation, we have derived organoids from small intestines from WT and FXR−/− mice, as described in^17^. We show that GW4064 and MF reduced *Tnfα* and *Cxcl2* expression only in WT but not in FXR−/− organoids (Fig. 3C). Notably, MF also showed FXR-independent decreases in *Tnfα* and *Cxcl2* expression, presumably via activating other NRs such as glucocorticoid receptor (GR). Also in concurrence with the HepG2 model system, in WT but not in FXR−/− organoids treated with GW4064, direct FXR target gene expression of *Shp*, *Fgf15* and *Ibabp* is increased, which is absent or decreased upon MF stimulation (Fig. 3D), indicating that the effect is mediated by FXR. These data independently confirm that compared to GW4064, MF has equal capacity to inhibit NF-κB target gene expression, but not in regulating direct FXR target genes. This suggests that MF is a gene selective FXR modulator.

### Computational studies reveal three putative binding modes of mometasone furoate to FXR

**Figure 4:**
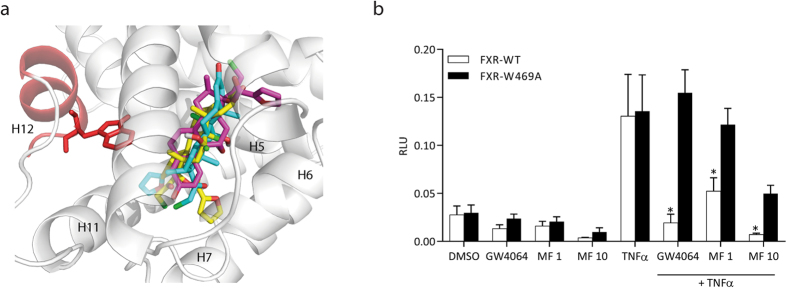
Superposition of the three predicted MF binding modes. Three different binding modes of MF to FXR, as determined by docking studies, are depicted. (**A**) Binding mode 1 suggests that the furoate group of MF is buried into the FXR binding site pointing towards helix 7 (yellow carbons); binding mode 2 is characterized by the furoate group of MF oriented toward helix 11 and helix 12 (blue carbons); binding mode 3 is head-to-tail flipped with respect to binding modes 1 and 2 with the furoate group of MF oriented toward the helix 5 and 6 (magenta carbons). Helix 12 is shown in red. (**B**) HEK293T cells transfected with NF-κB reporter, expression plasmids for wild type FXR (FXR-WT) or mutant FXR (FXR-W469A) and RXR, and pTK-renilla construct were treated with DMSO, 1 μM GW4064, 1 or 10 μM MF as indicated, or TNFα, for 24 hours. The NF-κB luciferase reporter assay was performed in quadruplicate. *p < 0.001 compared to FXR-W469A cells treated with GW4064 or MF plus TNFα.

Docking calculations and molecular dynamic simulations were carried out to explore the putative binding mode of MF to FXR. Since twenty-five agonist-bound FXR LBD co-crystal structures are currently available on RCSB Protein Data Bank^18^, we decided to use Phase Shape Screening to identify the FXR crystal structure with the suitable conformation able to accommodate MF. Therefore, all the available crystallographic structures of FXR agonists were screened using MF as query structure to determine the highest pharmacophore-based shape similarity between the compound and co-crystallized ligands. OCA^19^ was found to be the ligand endowed with the greater shape similarity. Accordingly, the co-crystal structure of FXR LBD in complex with OCA (pdb code: 1OSV^20^) was selected for the docking calculations. MF was flexibly docked into FXR LBD using Glide software. Ten docking solutions were retrieved and clustered into three different binding modes (clustering criterion: RMSD < 2 Å). Three poses, namely “binding mode 1–3”, representative for each cluster, were selected based on the best docking score (XP Gscore; see Table 1). Binding mode 1 represents the energetically favored docking pose (XP Gscore −10.02) and the most represented binding mode, characterized by the furoate group buried inside the FXR binding site establishing positive hydrophobic contacts with helix 7 (Fig. 4A). Binding mode 2 is similar to binding mode 1. In this case, MF orientation in the FXR binding cavity is rotated approximately 90° pointing the furoate group towards a region between helix 11 and helix 12 (Fig. 4A). The less represented binding mode 3 has a low binding score compared to binding modes 1 and 2 (Table 1). The docking pose is head-to-tail flipped so that the MF steroid A ring points towards the core of the FXR LBD with the furoate group oriented towards the solvent in a region between helix 5 and helix 6 (Fig. 4A). It is interesting to note that the orientation of MF proposed by binding modes 1 and 2 is similar to that experimentally observed for MF when it binds to GR^21^ and progesterone receptor (PR)^22^.

To support the computational studies, we performed a luciferase assay in which we compared NF-κB transcriptional activity of wild type FXR (FXR-WT) with mutant FXR (FXR-W469A). The mutated amino acid is located in helix 12, a crucial portion of the FXR LBD involved in the ligand-dependent cofactor recruitment. Upon GW4064 and MF stimulation, FXR-W469A showed significantly reduced transrepression activity compared to FXR-WT, suggesting that MF action is dependent upon the ligand binding domain of FXR (Fig. 4B).

**Figure 5:**
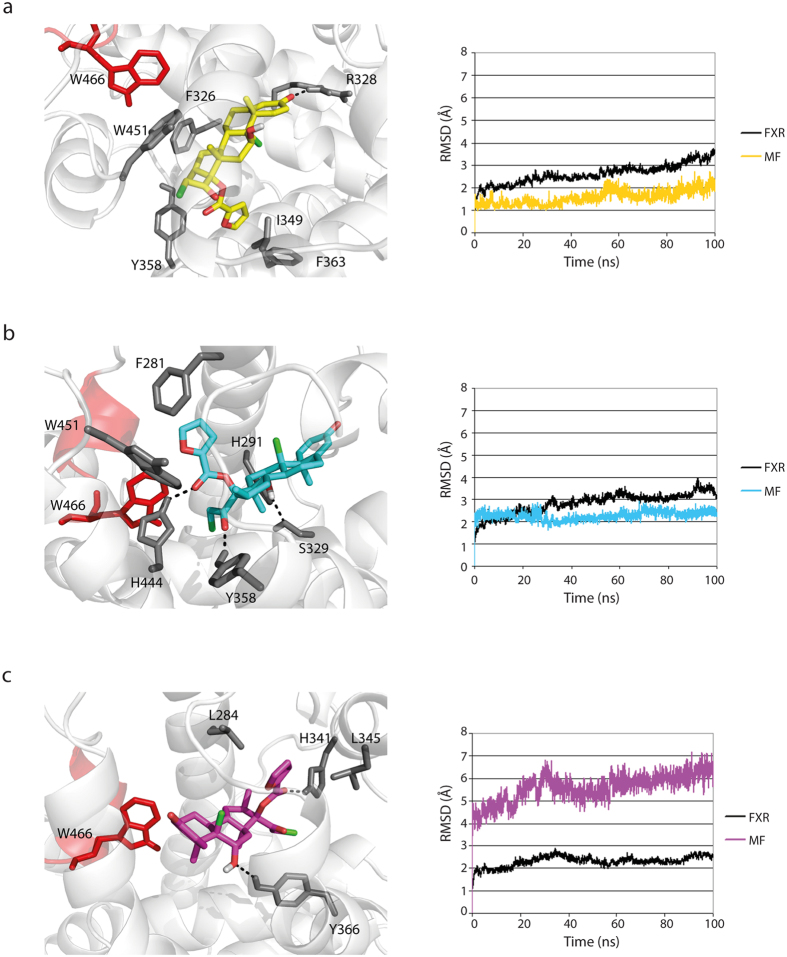
Graphical representation of the MF-FXR interactions. Graphical representation of the MF-FXR interactions and RMSD calculated during 100 ns of molecular dynamic simulations of the three binding modes suggested by docking studies. (**A**) binding mode 1 is stabilized mainly by hydrophobic interactions; (**B**) binding mode 2 is the most stabilized complex due to the high number of both hydrophobic and hydrophilic contacts conserved; (**C**) binding mode 3 is the less stabile binding mode despite conserved interactions are established during the simulation.

Next, we assessed the stability of the observed binding modes performing 100 ns of molecular dynamic simulation for the three selected MF/FXR docking complexes. Binding mode 1 was found to be maintained over the simulation time suggesting a high stabilization (MF average RMSD with respect to the starting conformation of 1.59 ± 0.30 Å; Fig. 5A). Moreover, we observed that i) the MF steroid core establishes good hydrophobic contacts with the side chains of Trp451 and Phe326, ii) the furoate group is oriented towards helix 7 thus positively interacting with side chains of Tyr358, Phe363 and Ile349, iii) the carbonyl group at C3 position engages an hydrogen bond with Arg328 side chain (Fig. 5A). A greater stabilization was observed during the molecular dynamic simulation of binding mode 2. Here, MF undergoes a slight conformational adjustment during the first picoseconds of the simulation until reaching a more stable conformation that is maintained throughout the simulation time (MF average RMSD with the respect to the starting conformation of 2.26 Å ± 0.20 Å) (Fig. 5B). Accordingly, binding mode 2 is stabilized by both hydrophobic and polar interactions defined as follows: i) particularly stable hydrogen bonds between the hydroxy group at the C11 *β*-position and the side chains of Ser329 and His291, ii) hydrogen bonds between carbonyl groups of C17 substituents and Tyr358 and His444 side chains, iii) π-π and hydrophobic interactions of the furoate group with the aromatic side chains of Phe281, Trp451 and Trp466 (Fig. 5B). Therefore, giving the high number of the established favorable contacts, the interaction energy between MF and FXR during the molecular dynamic simulation of binding mode 2 was slightly greater than those determined for the simulation of binding mode 1 (binding mode 1 interaction energy = −73.16 ± 6 kcal/mol; binding mode 2 interaction energy = −81.83 ± 3.86 kcal/mol). Analysis of the molecular dynamic simulation of MF binding mode 3 revealed marked MF conformational changes for the first picoseconds of simulation during which MF is shifted deeper into the binding site. This movement is reflected in an average RMSD from the starting conformation of 5.71 Å ± 0.50 Å (Fig. 5C). Moreover, MF undergoes further conformational adjustments for the whole simulation time, suggesting a lesser degree of stabilization of this complex despite the favorable protein-ligand interaction energy (−75.94 ± 5.48 kcal/mol). During the simulation, the C11 β-OH group of MF was found to interact stably with the polar side chain of Tyr366 through a hydrogen bond while the furoate carbonyl moiety is involved in an additional hydrogen bond interaction with the side chain of His341. In addition, the ring A of the steroid scaffold engages Trp466 side chain in stable hydrophobic contacts and the furoate group establishes good hydrophobic interactions with the side chains of Leu284 and Leu345.

### GW4064 and MF reduce p65 recruitment to pro-inflammatory gene promoters

**Figure 6:**
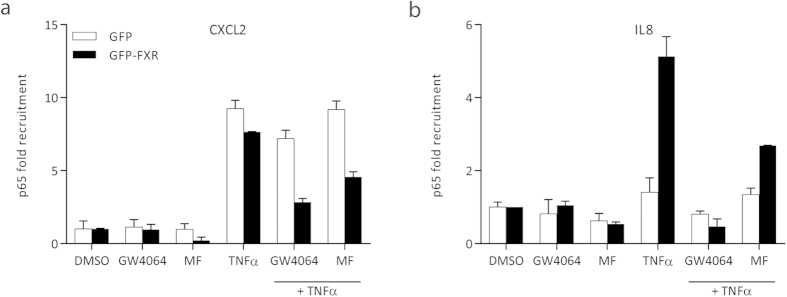
GW4064 and MF reduce p65 recruitment to pro-inflammatory gene promoters. HepG2-GFP and HepG2-GFP-FXR cells were treated with DMSO, GW4064, MF, and TNFα, as indicated. Cross-linked chromatin from these cell lysates was precipitated using an anti-p65 antibody, and analyzed by qPCR with primers specific to the known p65 binding regions of *CXCL2* and *IL8*.

Upon an inflammatory stimulus, NF-κB subunits translocate to the nucleus and bind to target regions in the genome, leading to activation of target genes. To investigate whether recruitment of NF-κB to promoters is altered upon MF and GW4064 stimulation, we performed chromatin IPs for p65, the main activating NF-κB subunit, in HepG2-GFP and HepG2-GFP-FXR cells (Fig. 6). We show that at 4hours after GW4064 and MF stimulation, p65 binding to the *CXCL2* (Fig. 6A) and *IL8* (Fig. 6B) promoters is reduced in HepG2-GFP-FXR cells, but not in HepG2-GFP cells. Control regions (*SHP* and *β-hemo* promoters) showed no p65 recruitment (data not shown). We have previously shown that FXR binds p65 in GST-pull down experiments^23^. We propose that this interaction with FXR prevents p65 binding to the *IL8* and *CXCL2* promoters, leading to the observed reduction in transcriptional activation.

## Discussion

FXR is regarded as a promising drug target for many liver and gastrointestinal disorders. At present, FXR agonists are in Phase II and III clinical trials for nonalcoholic fatty liver disease (NAFLD), nonalcoholic steatohepatitis (NASH), primary sclerosing cholangitis (PBC), and primary biliary cirrhosis (PSC)^24^. We know that FXR regulates transcription of many target genes involved in BA, glucose and fat metabolism. To bypass potential side-effects from treatment, selective FXR modulation might be advantageous over full agonism. We hypothesized that gene-selective ligands may separate two different molecular mechanisms by which FXR functions, i.e. by direct DNA binding or by tethering to another transcription factor. Current knowledge suggests that FXR regulation of BA, glucose and fat metabolism involves direct FXR binding to promoter sequences. In contrast, FXR anti-inflammatory actions have been shown to occur via binding of FXR to NF-κB, thereby inhibiting its activity^9^,^10^,^14^. In order to identify FXR ligands that are able to inhibit NF-κB activity, we have set up an automated luciferase reporter assay. We tested 1,200 drugs using a high-throughput luciferase screen to determine their capacity to inhibit NF-κB. Of the 5 compounds which significantly reduced TNFα-induced NF-κB transcriptional activity, 3 compounds operated in an FXR-dependent manner: nicardipine hydrochloride, cilnidipine, and MF. Transactivation of SHP and IBABP promoters by MF was minimal compared to the full agonist GW4064. Our data confirm the recent study by Hsu *et al.,* who also identified nicardipine hydrochloride and cilnidipine as FXR modulators^16^. Since Hsu *et al.* identified these compounds by a co-activator recruitment assay, which is corresponding to the direct DNA binding of FXR, we did not pursue them further in this paper. Instead, our attention was directed towards MF because of its steroid-like structure, a scaffold that particularly fits with the LBD of FXR^25^. MF is a nanomolar glucocorticosteroid characterized by chlorine substituents at the C9α- and C21-position, endowed with antipruritic, anti-inflammatory, and vasoconstrictive properties. MF has not only high affinity for GR, but also for other NRs, including PR and mineralocorticoid receptor (MR)^22^,^26^. Like other corticosteroids, the anti-inflammatory efficacy of MF is mediated by the repression of inflammatory gene transcription either directly (via transrepression) or by activating transcription of anti-inflammatory/repressive factors (transactivation)^27^. Here, we show that MF binds to and inhibits NF-κB activity in a dose responsive and FXR-dependent manner, both in reporter assays and on endogenous pro-inflammatory genes in HepG2 cells stably overexpressing FXR and intestinal organoids derived from WT and FXR−/− mice. These results are comparable to the full FXR agonist GW4064. However, FXR target genes which are activated upon direct FXR binding to their promoters did show no or reduced expression in all model systems upon MF treatment. We therefore conclude that it is possible to separate FXR direct transactivation from FXR mediated tethered transrepression.

The FXR-mediated decrease in expression of pro-inflammatory genes by MF and GW4064 presumably does not involve the recruitment of co-activators, but rather involves binding of FXR to p65, thereby prohibiting its binding to promoters (Fig. 6 and references^11^,^23^). The results of the AlphaScreen assay (Fig. 2E) concur with this hypothesis, because incubation with MF results in low efficacy for the recruitment of the coactivator peptide of SRC-1 to the FXR-LBD as compared to CDCA (12%), while the effect on mRNA expression of pro-inflammatory genes is similar. Docking analysis and molecular dynamic simulations suggested three putative binding modes of MF to FXR: binding mode 1 is characterized by the furoate group buried inside the FXR binding site towards helix 7; binding mode 2, similar to binding mode 1, but rotated approximately 90°, where the furoate group is oriented in a region between helix 11 and helix 12; and binding mode 3, head-to-tail flipped pose with respect to binding modes 1 and 2, where the steroid A ring points towards the core of the FXR LBD and the furoate group is oriented towards the solvent in a region between helix 5 and helix 6. Molecular dynamics simulations of the three proposed MF binding modes revealed that binding mode 1 is stable over the simulation time as the result of several interactions, mainly hydrophobic. Binding mode 2 was found to be even more stable, indeed, the lower standard deviation in the RMSD values accounts for a greater stabilization of the ligand inside the binding site. Binding mode 2 was also found to be the slightly favored MF binding mode from an energetically point of view because of both hydrophobic and hydrogen bond interactions. Binding mode 3 showed slightly less stabilization in terms of MF conformation despite a good energy content due to the conserved polar and hydrophobic contacts established during the molecular dynamic simulation. Although not definitive, all together these results suggest that binding mode 2 may represent the most probable binding mode of MF to FXR, though we cannot exclude binding mode 1 and 3 as plausible alternative solutions.

Considering that MF is a clinical drug against persistent asthma as well as a well-established treatment for a variety of inflammatory corticosteroid-responsive dermatoses, such as chronic hand eczema, atopic dermatitis (AD), seborrhoeic dermatitis, and psorias^28^, our findings reveal an alternative template for design of FXR ligands with therapeutic potential to rapid clinical applications by providing a safe lead compound. Structural modifications on the MF scaffold are therefore particularly sought to increase FXR potency and selectivity. In conclusion, we show that MF selectively activates FXR anti-inflammatory actions. Although MF itself is not suitable to pursue as an FXR selective ligand, this opens an exciting new avenue for selective FXR agonism and future opportunities for treatment of chronic inflammation of liver and gut.

## Methods

### Materials

The Prestwick Chemical Library® containing 1,200 FDA/EMA approved drugs as 10 mM stock solutions in 96 wells plates was purchased from Prestwick (Prestwick Chemical, Illkirch, France). Cilnidipine (C), mometasone furoate (MF), nicardipine hydrochloride (NH), quinacrine dihydrochloride dehydrate (QDD), topotecan (T), GW4064, and CDCA were purchased from Sigma-Aldrich, TNFα and Prot A beads from Roche. Di(N-succinimidyl) glutarate (DSG), 97% was obtained from Synchem UG & Co. Antibody used for p65 (C-20) ChIP was bought from Santa Cruz.

### Cell and organoid culture

HEK293T cells were grown in Dulbecco’s modified Eagle’s medium (DMEM, Sigma-Aldrich), supplemented with 10% FCS and 1% penicillin/streptomycin (Sigma). HepG2 cells were cultured in DMEM supplemented with 10% FCS, 1% penicillin/streptomycin, and 1% L-glutamine (Lonza; Verviers, Belgium). Medium of HepG2 cells stably expressing pLenti-CMV-neo-GFP (HepG2-GFP) or pLenti-CMV-neo-GFP-FXR (HepG2-GFP-FXR) constructs was supplemented with 100 μg/μl G418. GFP-FXR was cloned in pLenti CMV Neo DEST (705-1), a gift from Eric Campeau (Addgene plasmid #17392)^29^. Virus production and transduction of HepG2 cells was done following standard procedures. Small intestines (SI) were isolated from 3WT and 3FXR−/− mice, washed with cold PBS, the epithelial layer gently scraped. The remaining tissue with intact crypts was washed several times with cold PBS and subsequently incubated with 5 mM EDTA/PBS for 1 hour to isolate crypt cells as previously described^17^. The crypt cells were embedded in matrigel and seeded in 24 well plates containing advanced DMEM/F12 supplemented with penicillin/streptomycin, HEPES, Glutamax, Wnt, n-Acetyl-cysteine, growth factors (Noggin, R-spondin, mEGF), B27, Y-27632, A83-01 and p38 inhibitor.

### Automated high-throughput luciferase reporter assay

We screened the Prestwick Chemical Library® using a luciferase reporter assay. HEK293T cells were bulk transfected with a NF-κB responsive element reporter construct (pGL2-2κB), pcDNA3.1-FXRα2 or pcDNA3.1-FXRα2-W469A, and pcDNA3.1-RXRα expression plasmids, and pTK-renilla as an internal transfection control. The next day, cells were transferred to poly-l-lysine (Sigma-Aldrich) coated 384-well plates (Corning) using Multidrop™ Combi Reagent Dispenser (Thermo Scientific). Cells were stimulated in triplicate with vehicle (DMSO), GW4064 (1 μM), TNFα (5 ng/ml), or with the Prestwick Chemical Library® (10 μM) in presence of TNFα for 24 hours using the Caliper Sciclone liquid handling robot, followed by cell lysis, and firefly and renilla luciferase measurement (Dual-Luciferase®Reporter AssaySystem; Promega) using a SpectraMax® M5e Multi-Mode Microplate Reader (Molecular Devices).

To determine transactivation, HEK293T cells were transfected with either pGL2-SHP or pGL3-IBABP promoter constructs in combination with pcDNA3.1-FXRα2, pcDNA3.1-RXRα, and pRL-CMV renilla plasmids. Cells were stimulated with vehicle (DMSO), GW4064 or MF for 24 hours. Subsequently, cells were lysed and luciferase activity was determined.

### Data normalization and hit selection

The Prestwick library consisted of four 384-wells plates. Each library plate was assayed in triplicate. Data were normalized to the average TNFα value of the corresponding library plate. To perform hit selection, the Manhattan distance score for each drug and control value for each individual library plate was calculated against the average score of the TNFα values. This calculation was based on RLU values, i.e. the luciferase/renilla ratio. The distance scores were transformed into p-values using the Cumulative Distribution Function. Hits were considered statistically significant if p ≤ 0.05. Only drugs that significantly inhibited NF-κB transcriptional activity were considered for further analysis. Subsequently, drugs showing low renilla activity, suggesting drug cytotoxicity and/or low transfection efficiency, were eliminated.

### mRNA isolation and quantitative RT-PCR

HepG2-GFP and HepG2-GFP-FXR cells and SI organoids were treated with vehicle (DMSO), 1 μM GW4064, 5 ng/ml TNFα, TNFα plus GW4064, or 10 μM MF in the presence or absence of TNFα for 24 hours. RNA was isolated using Trizol® reagent (Ambion/Life Technologies). RNA was reverse-transcribed using Superscript II (Invitrogen) according to manufacturer’s protocol. Quantitative RT-PCR was performed using Fast start Universal SYBR Green Master mix (Roche) and primers for FXR target genes on CFX384™ Real-Time system (Biorad). Target gene expression was normalized to housekeeping gene β2-microglobulin (HepG2) or cyclophilin A (organoids). Primers are listed in Table 2. Data are presented as fold change.

### Co-factor recruitment assay

FXR co-factor recruitment was assayed using the AlphaScreen technology according to manufacturer’s instructions. In brief, GST-tagged FXR-LBD was coupled to anti-GST-acceptor beads, and biotinylated-SRC-1 peptide (co-activator) to streptavidin donor beads. Presence of ligand induces a conformational change of the LBD, followed by co-activator binding. Upon illumination at 680 nm, energy is transferred from the donor to acceptor beads, generating a luminescent signal. Biotinylated SRC-1, GST-FXR and ligand were incubated for 1 hour. Detection mix (donor and acceptor beads) was added, followed by 4 hour incubation. Reading was performed using Envision®Multilabel Reader (Perkin Elmer). Dose response curves were performed in triplicate and EC_50_ values were determined.

### Docking studies and molecular dynamics simulations

Chemical structure of MF was drawn using Maestro building fragment tool (Maestro, version 9.3, Schrödinger, LLC, New York, NY, 2012). LigPrep software (LigPrep, version 2.5, Schrödinger, LLC, New York, NY, 2012) was used to generate the three-dimensional structure. The correct chirality of the compound was assessed and the ionization states at pH 7 ± 2 were calculated. The twenty-five deposited FXR co-crystal structures were downloaded from RCSB Protein Data Bank^18^ and the co-crystalized ligands were used to perform a Phase Shape Screening (Phase, version 3.4, Schrödinger, LLC, New York, NY, 2012) against MF. Phase Shape Screening was run in a pharmacophore-based mode indicating OCA^19^ as the most similar compound to MF with respect to the shape. The co-crystal structure of FXR in complex with OCA (pdb code: 1OSV^20^) was therefore selected for docking studies and submitted to the Protein Preparation Wizard workflow (Maestro, version 9.3, Schrödinger, LLC, New York, NY, 2012). The receptor grid was then calculated using Glide (Glide, version 5.8, Schrödinger, LLC, New York, NY, 2012) software. The centroid of the co-crystalized ligand was taken as the center of the grid and the docking space was set as 29 Å cubic box. MF was then docked in a stepwise manner: MF was flexibly docked into the prepared grid using the Glide Standard Precision (SP) algorithm. Ten poses were collected and subsequently refined with the more accurate Extra Precision (XP) algorithm. The retrieved docking solutions were then clustered into three distinct binding modes (clustering criterion: root-mean-square deviation (RMSD) <2 Å). Three poses representative for the three predicted binding modes, were selected relying on the best Glide XP Gscore and submitted to molecular dynamic simulations using Desmond (Desmond Molecular Dynamics System, version 3.6, D. E. Shaw Research, New York, NY, 2013, Maestro-Desmond Interoperability Tools, version 3.6, Schrödinger, New York, NY, 2013). The systems were built by solvating each complex with SPC water solvent and neutralized by adding sodium ions. The periodic boundary conditions have been set defining a 10 Å width orthorhombic simulation box. The temperature of the system was set to 300 K. The simulations were performed in NPT ensemble using Nose-Hoover chain thermostat and Martyna-Tobias-Klein barostat. The force field used was OPLS2005. Systems were relaxed first and subsequently submitted to 100 ns of trajectory production. Molecular dynamics trajectories were analyzed with the Simulation Event Analysis and the Simulation Interaction Diagram implemented in Desmond. Average RMSD and interaction energies were calculated excluding the first 10 ns of stabilization of the systems. Figures were prepared using PyMOL (http://www.pymol.org).

### ChIP-qPCR

To determine p65 recruitment to target promoters, HepG2-GFP and HepG2-GFP-FXR cells were treated with vehicle (DMSO), 1 μM GW4064, 10 μM MF or 5 ng/ml TNFα, as indicated for 4 hours. ChIP was performed according to Saccani *et al.*^30^, with a two-step crosslinking using DSG and formaldehyde, as described in Nowak *et al.*^31^. qPCR was performed as described above. Data are presented as fold change relative to DMSO. Primers are listed in Table 2.

### Data analysis

GraphPad Prism software version 6.02 (GraphPad Software, Inc.) was used for figure preparation, determining EC_50_ and IC_50_ values, and statistical analysis. Unpaired t-test, two-tailed, was used for luciferase assay. For luciferase assay and qRT-PCR results, each bar represents mean ± SD, or mean ± SEM (organoids). P-values ≤ 0.05 were considered statistically significant.

## Additional Information

**How to cite this article**: Bijsmans, I. T. G. W. *et al.* The glucocorticoid mometasone furoate is a novel FXR ligand that decreases inflammatory but not metabolic gene expression. *Sci. Rep.* **5**, 14086; doi: 10.1038/srep14086 (2015).

## Figures and Tables

**Table 1:** Summary of the docking calculation of MF into FXR LBD.

Cluster of docking solutions	No of poses in cluster	Top XP Gscore (kcal/mol)	RMSD (Å) versus top ranked solution
Binding mode 1	6	−10.02	0
Binding mode 2	3	−8.61	4.41
Binding mode 3	1	−8.46	8.50

**Table 2:** Primer sequences.

Gene	Forward primer (5′-3′)	Reverse primer (5′-3′)
hB2M	GGCTATCCAGCGTACTCCAAA	CGGCAGGCATACTCATCTTTTT
hCXCL2	CCCATGGTTAAGAAAATCATCG	CTTCAGGAACAGCCACCAAT
hICAM1	CCTTCCTCACCGTGTACTGG	AGCGTAGGGTAAGGTTCTTGC
hFGF19	CGTGCGGTACCTCTGCAT	TCTCCTCCTCGAAAGCACA
hIL8	GGAAGGAACCATCTCACTGTG	GGGTGGAAAGGTTTGGAGTA
hKNG1	AGTAAAACGGGCCCAAAGAC	TCGTTTGCACAATTGAGTAGGT
hMCP-1	CAGCCAGATGCAATCAATGCC	TGGAATCCTGAACCCACTTCT
hSDC1	AGGATGGAGGTCCTTCTGC	CCGAGGTTTCAAAGGTGAAGT
hSHP	AGGGACCATCCTCTTCAACC	TTCACACAGCACCCAGTGAG
mCxcl2	AAAATCATCCAAAAGATACTGAACAA	CTTTGGTTCTTCCGTTGAGG
mCyclophilin A	GGAGATGGCACAGGAGGAA	GCCCGTAGTGCTTCAGCTT
mFgf15	AAAACGAACGAAATTTGTTGGAA	ACGTCCTTGATGGCAATCG
mIbabp	TTGAGAGTGAGAAGAATTACGATGAGT	TTTCAATCACGTCTCCCTGGAA
mShp	CGATCCTCTTCAACCCAGATG	AGGGCTCCAAGACTTCACACA
mTnfα	ACGGCATGGATCTCAAAGAC	AGATAGCAAATCGGCTGACG
hCXCL2 promoter	ATGGTTGGGGCTGGAAAG	CGCCTTCCTTCCGAACTC
hIL8 promoter	CATCAGTTGCAAATCGTGGA	AGAACTTATGCACCCTCATCTTTT
